# Changes in recall rate, biopsy rate and cancer detection since the introduction of digital mammography

**DOI:** 10.1186/bcr3273

**Published:** 2012-11-09

**Authors:** TG Fyall, CRM Boggis, SM Astley, JC Sergeant, J Morris, S Whiteside, M Wilson

**Affiliations:** 1 The University of Manchester, UK

## Introduction

Analogue mammography is gradually being replaced by digital mammography for breast screening. Digital mammography has been shown to be at least as good as analogue mammography at cancer detection, but the picture regarding other outcomes such as recall rate and biopsy rate is less clear.

## Methods

Outcome data from 62,599 routine screening mammograms taken in Greater Manchester between January 2010 and March 2012 were gathered from screening records. Mean recall, biopsy/cytology and cancer detection rates were calculated for analogue and digital screening and compared. After complete conversion to digital screening, the same three measures were obtained on a monthly basis over 2 years. The data were analysed to identify any trends in the measures.

## Results

The digital mammography recall rate was 4.69% (2,505/53,444), with analogue significantly lower at 3.58% (328/9,155) (*P *< 0.001). The digital biopsy/cytology rate was 1.88% (1,005/53,444), with analogue significantly lower at 1.49% (136/9,155) (*P *= 0.01). The cancer detection rate (per 1,000 screens) was 6.74 (56/9,155) for digital mammography and 6.15 (358/53,444) for analogue; however, this difference was not significant (*P *= 0.53). The recall rate showed a strong positive correlation with time (*r *= 0.71, *P *< 0.001) and the biopsy rate showed a weak positive correlation (*r *= 0.52 *P *= 0.011). Cancer detection showed a weak negative correlation with time (*r *= -0.567 *P *= 0.05).

## Conclusion

The introduction of digital mammography resulted in higher recall and biopsy rates, but no change in cancer detection. Recall and biopsy rates, whilst still well within recommended levels, appear to be increasing since the introduction of digital mammography; this indicates a need for regular monitoring.

